# Anatomy of the external branch of the superior laryngeal nerve in Asian population

**DOI:** 10.1038/s41598-017-15070-9

**Published:** 2017-11-02

**Authors:** Yong Hoon Cha, Seo-Young Moon, O. Jehoon, Tanvaa Tansatit, Hun-Mu Yang

**Affiliations:** 1 Department of Oral and Maxillofacial Surgery, Yonsei University College of Dentistry, Seoul, Korea; 2 Department of Anatomy, Yonsei University College of Medicine, Seoul, Korea; 3 The Chula Soft Cadaver Surgical Training Center, Department of Anatomy, Faculty of Medicine, Chulalongkon University, Bangkok, Thailand

## Abstract

Injury to the external branch of the superior laryngeal nerve (eSLN) can cause a hoarse or weak voice with dysergia of the cricothyroid. The present study provided the topographic information of the eSLN in the Asian and verified anatomical validity of the landmarks previously recruited to localize the eSLN. Thirty specimens were dissected from 16 human embalmed cadavers (12 men and four women; mean age: 80.5 years). The vertical distance between the eSLN and the apical pole of the thyroid gland (AP) was 8.2 ± 4.2 mm. It descended over the AP with <1 cm distance in 51.7%, >1 cm distance in 27.6% and under the AP in 20.7%. The piercing point (PP) of the eSLN to the muscles located 26.0 ± 5.5 mm posterior and 14.7 ± 5.0 mm inferior to the laryngeal prominence. Generally, the PP located superoposterior to the midpoint of the joint between the joint of inferior constrictor and cricothyroid (ICJ). The distance between the PP and the midpoint was 8.7 ± 5.1 mm. We found that 1) the Asian had the eSLN located over the AP with <1 cm distance about half cases, 2) the PP can be a consistent reference for the eSLN identification, 3) the ICJ can be a useful landmark to preserve the eSLN at the PP.

## Introduction

The musculature and mucosa of the larynx play a key role in vocalization, respiration, and crucial glottic reflexes associated with deglutition, coughing, and vomiting^1^. The vagus nerve branches into the superior laryngeal nerve (SLN) and recurrent laryngeal nerve (RLN). The SLN bifurcates into the internal branch, which conveys afferent fibers from the laryngeal mucosa, and the external branch (eSLN), which provides the only efferent fibers to the cricothyroid muscle (CT)^2^. These efferent fibers participate in meticulous control of the voice.

Great clinical emphasis has been placed on the topography of the RLN, though eSLN anatomy is also of great clinical significance. Iatrogenic injury to the eSLN occurs during improper dissection and ligation of adjacent vessels (e.g., superior thyroid artery, STA) in up to 58% of patients undergoing thyroid surgery. Dysergia of the CT secondary to eSLN injury can cause postoperative complications such as a hoarse, weak voice, vocal fatigue, or reduced vocal frequency^2–8^.

Despite its clinical importance, routine identification of eSLN anatomy has been hampered by topographic variation and difficulty in landmark recruitment. The STA has been regarded as a primary structure for the preservation and identification of the eSLN; however, it is difficult to provide precise metric information regarding the topographic relationship of the eSLN to the STA. While some authors have recruited the apical pole of the thyroid gland (AP) as a landmark for identification of the eSLN, the positional consistency of the AP must also be anatomically verified, as physiologic and pathologic conditions can alter AP position. Furthermore, previous studies have reported that the rate of eSLN identification varies considerably, ranging from 33% to 93%^7,8^. Friedman *et al*. reported that the eSLN pierced the superior fibers of the inferior pharyngeal constrictor (IPC), and that the course of the eSLN toward the CT was covered by the IPC^8^. Therefore, the position of eSLN entrance to the IPC may be useful for determining the course of the eSLN. In addition, a consistent landmark on the anterior side of the thyroid cartilage may allow for safe anterior approach of the CT after reflection of the strap muscles (e.g., sternothyroid muscle).

Meticulous verification of previous landmarks for the eSLN must be performed to facilitate the identification of the eSLN. The aim of the present study was to provide topographic information regarding the course of the eSLN with reference to the STA, AP, and CT in the Asian population, and to verify the reliability of these landmarks for localization of the eSLN. We also analyzed differences in eSLN topography between Asian specimens of the present study and Caucasian specimens described in previous studies.

## Results

**Figure 1:**
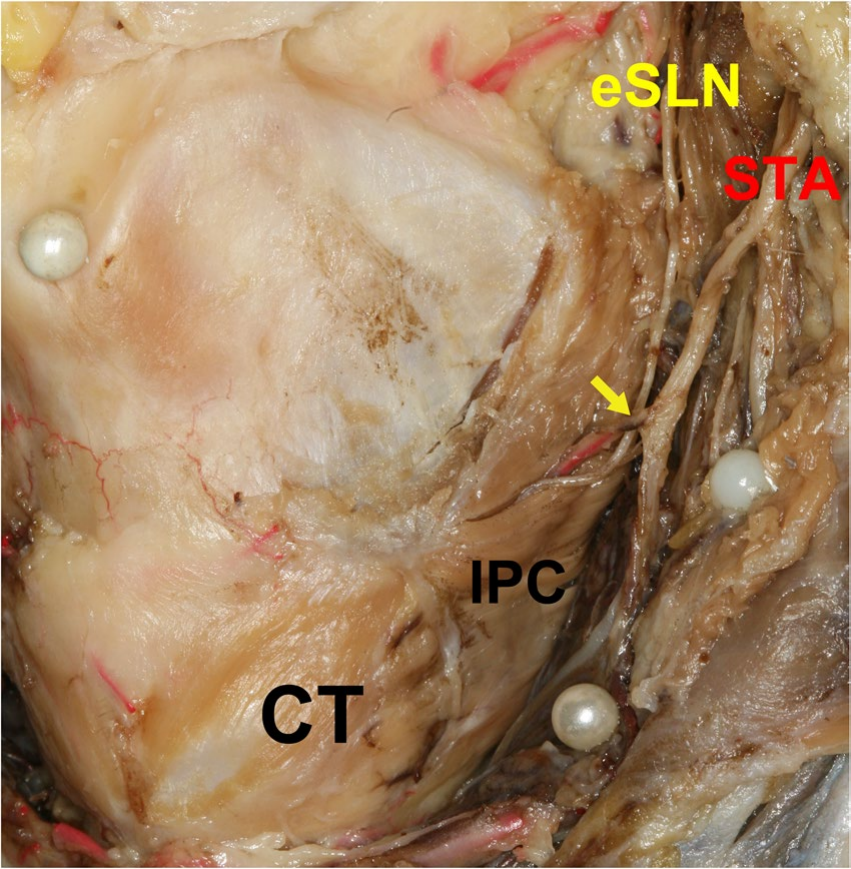
Arterial twig crossing the external branch of the superior laryngeal nerve (eSLN) from the superior thyroid artery (STA). Arrow indicates an STA twig crossing the STA. IPC, inferior pharyngeal constrictor; CT, cricothyroid.

The eSLN was identified in all but one case, medially juxtaposed to the STA as it proceeded in the anteroinferior direction. Tiny vessels from the main trunk of the STA crossed the eSLN in five cases (17.2%, Fig. 1). In most specimens, the eSLN exhibited a distinct trunk pattern (26 cases, 89.7%), whereas a plexiform pattern was observed in three specimens.

**Figure 2:**
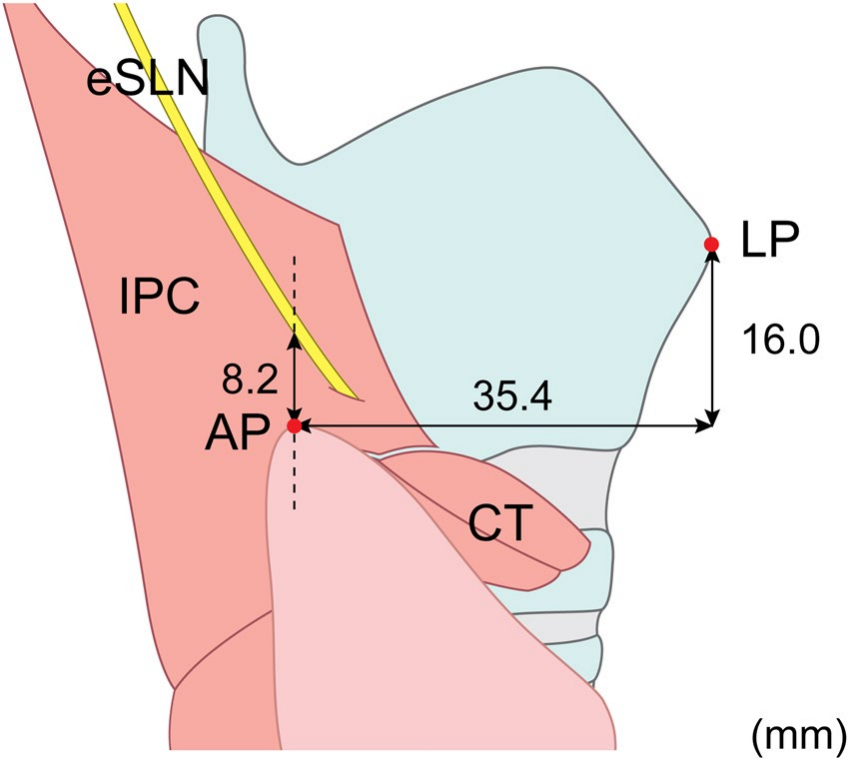
The position of the apical pole (AP) of the thyroid gland and the external branch of the superior laryngeal nerve (eSLN) into the laryngeal muscle. LP, laryngeal prominence; IPC, inferior pharyngeal constrictor; CT, cricothyroid.

The AP was located 35.4 ± 7.6  mm posterior and 16.0 ± 10.6 mm inferior to the LP, while its vertical distance from the eSLN was 8.2 ± 4.2 mm (Fig. 2). No significant differences in distance were observed between specimens from Korean and Thai individuals.

**Figure 3:**
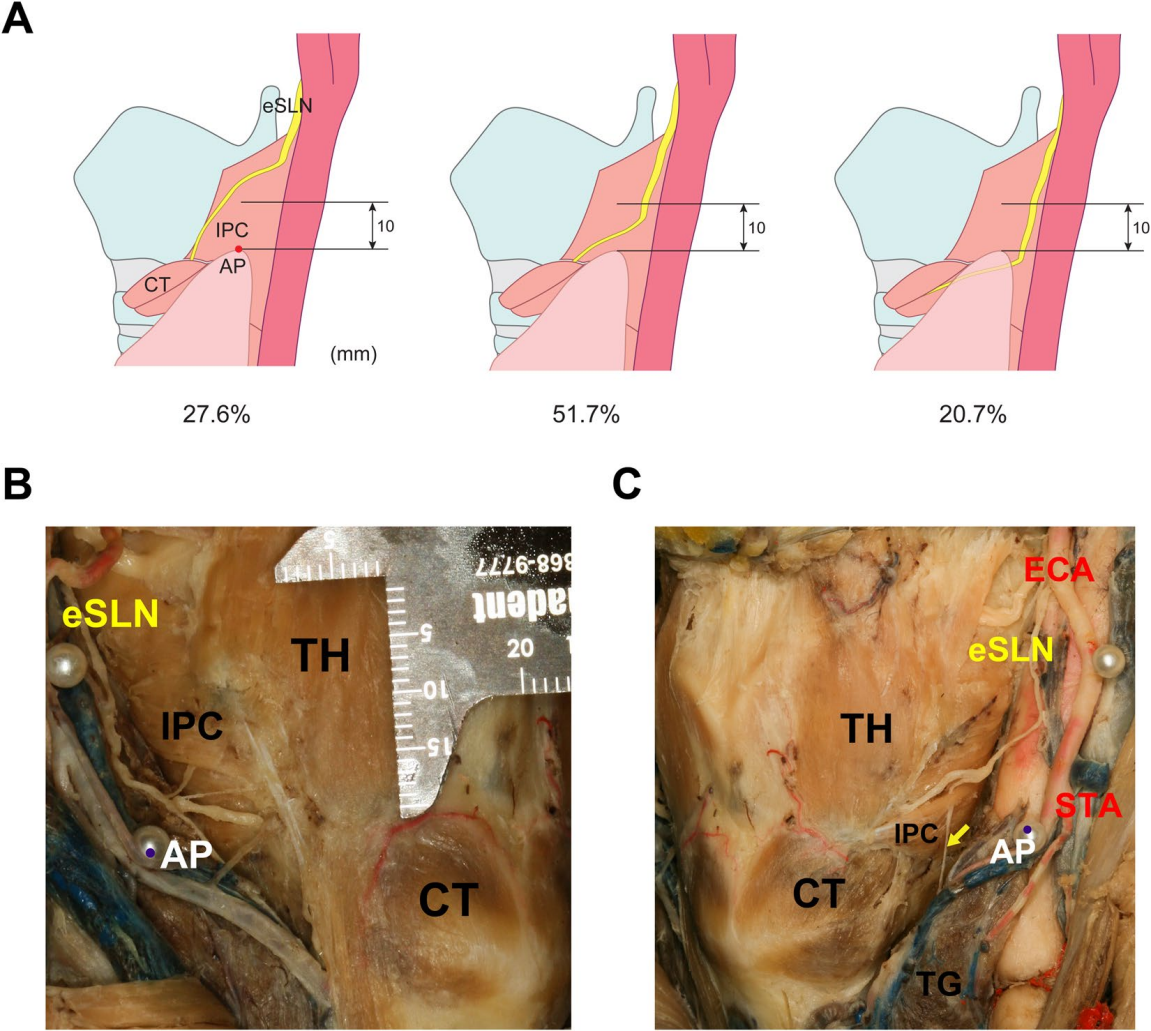
Topographic relationship between the external branch of the superior laryngeal nerve (eSLN) and the apical pole (AP) of the thyroid gland. (**A**) Three topographic patterns of eSLN position with reference to the AP. (**B**) In the most common pattern, the distance between the eSLN and AP was <1 cm. (**C**) Glandular twig (arrow) of the eSLN to the AP. IPC, inferior pharyngeal constrictor; CT, cricothyroid; TH, thyrohyoid; ECA, external carotid artery; STA, superior thyroid artery; TG, thyroid gland.

The proceeding pattern of the eSLN according to distance from the AP is categorized in Fig. 3B and Table 1. The eSLN descended less than 1 cm from the AP in 15 cases (51.7%), and this represented the most common pattern in both Korean and Thai samples (Fig. 3B). The eSLN descended more than 1 cm from the AP in eight cases (27.6%), and this pattern was observed in approximately one quarter of both Korean and Thai samples. In the remaining six cases (20.7%), the eSLN descended inferior to the AP as it proceeded deeply into the thyroid gland. Interestingly, there was no significant correlation between the vertical distance between the AP and LP, or the vertical distance between the AP and eSLN (*p* > *0.05*), despite large vertical deviation of the AP (*SD*: 10.6 mm). In 15 cases (51.7%), small twig-like ramifications branched from the main trunk of the SLN and entered the AP (Fig. 3C).

**Table 1:** Three topographic patterns based on distance between the external branch of the superior laryngeal nerve (eSLN) and the apical pole (AP) of the thyroid gland.

Vertical location of the eSLN		Korean specimens	Thai specimens	All specimens
Over the AP	>1 *cm Distance*	*27.8%*	*27.3%*	*27.6%*
	<1 *cm Distance*	*55.5%*	*45.4%*	*51.7%*
Under the AP		*16.7%*	*27.3%*	*20.7%*
Total		*100%*	*100%*	*100%*

**Figure 4:**
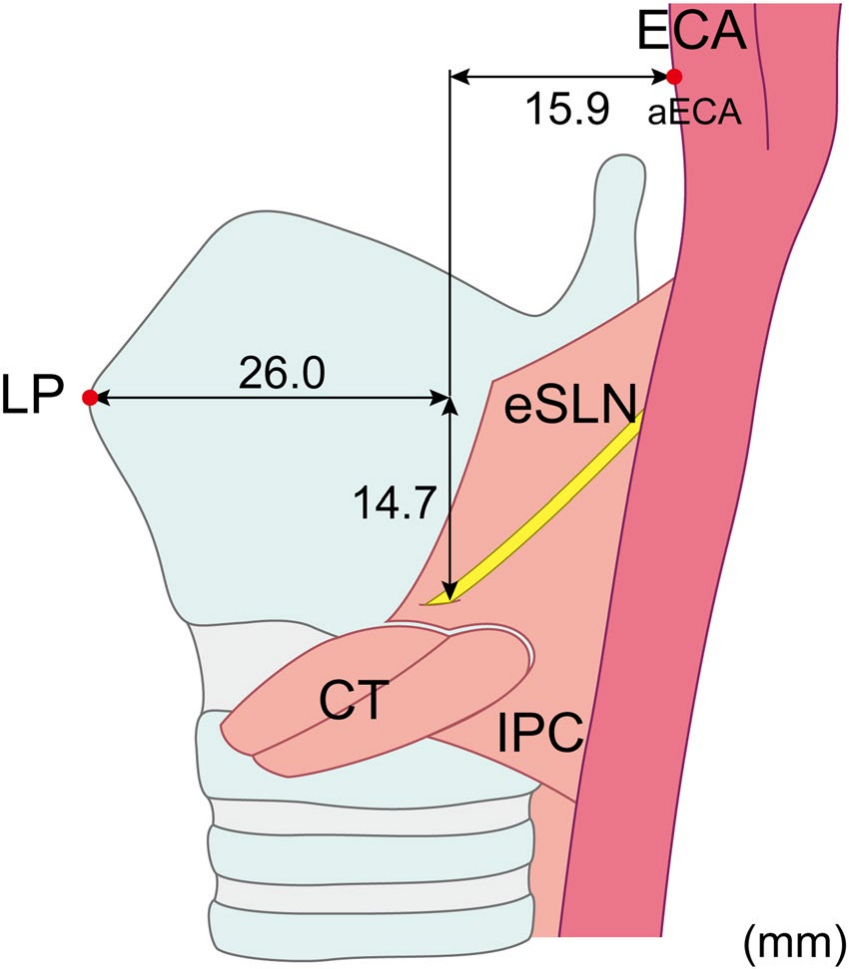
Location of the piercing point of the external branch of the superior laryngeal nerve (eSLN) with reference to the laryngeal prominence (LP) and the anterior border (aECA) of the external carotid artery (ECA). IPC, inferior pharyngeal constrictor; CT, cricothyroid.

The PP was located 15.9 ± 5.6 mm anterior to the aECA (Fig. 4). The horizontal position of the eSLN with reference to the aECA differed significantly between Korean and Thai specimens (*p* < 0.05): The eSLN was located more anteriorly to the aECA in Korean specimens (17.9 mm) than in Thai specimens (13.6 mm). The PP was located 26.0 ± 5.5 mm posterior and 14.7 ± 5.0 mm inferior to the LP. The horizontal distance between the LP and PP was greater in specimens obtained from men (27.5 mm) than in those obtained from women (22.5 mm) (*p* < 0.05).

**Figure 5:**
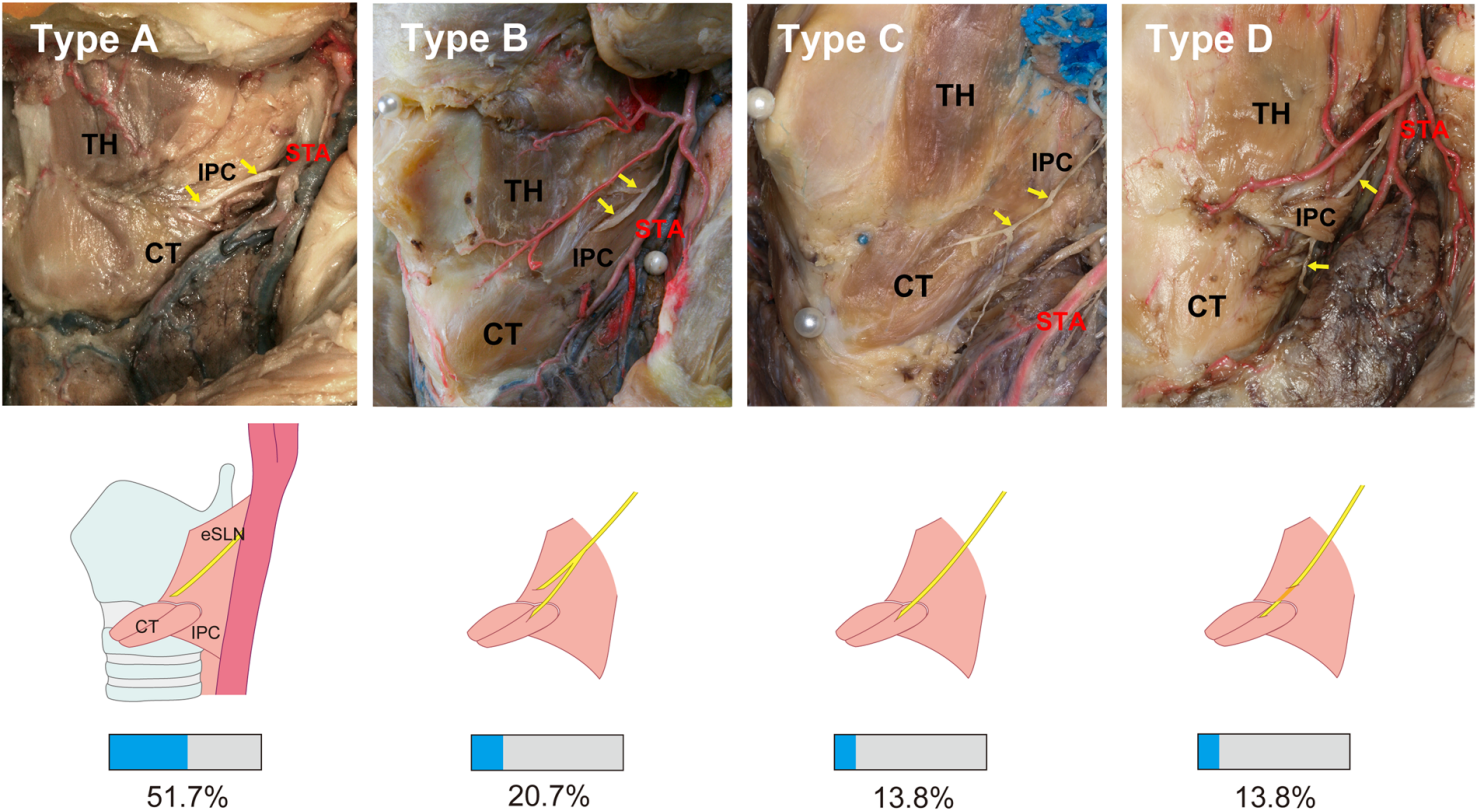
Four patterns of innervation for the external branch of the superior laryngeal nerve (eSLN). IPC, inferior pharyngeal constrictor; CT, cricothyroid; TH, thyrohyoid muscle; STA, superior thyroid artery.

**Figure 6:**
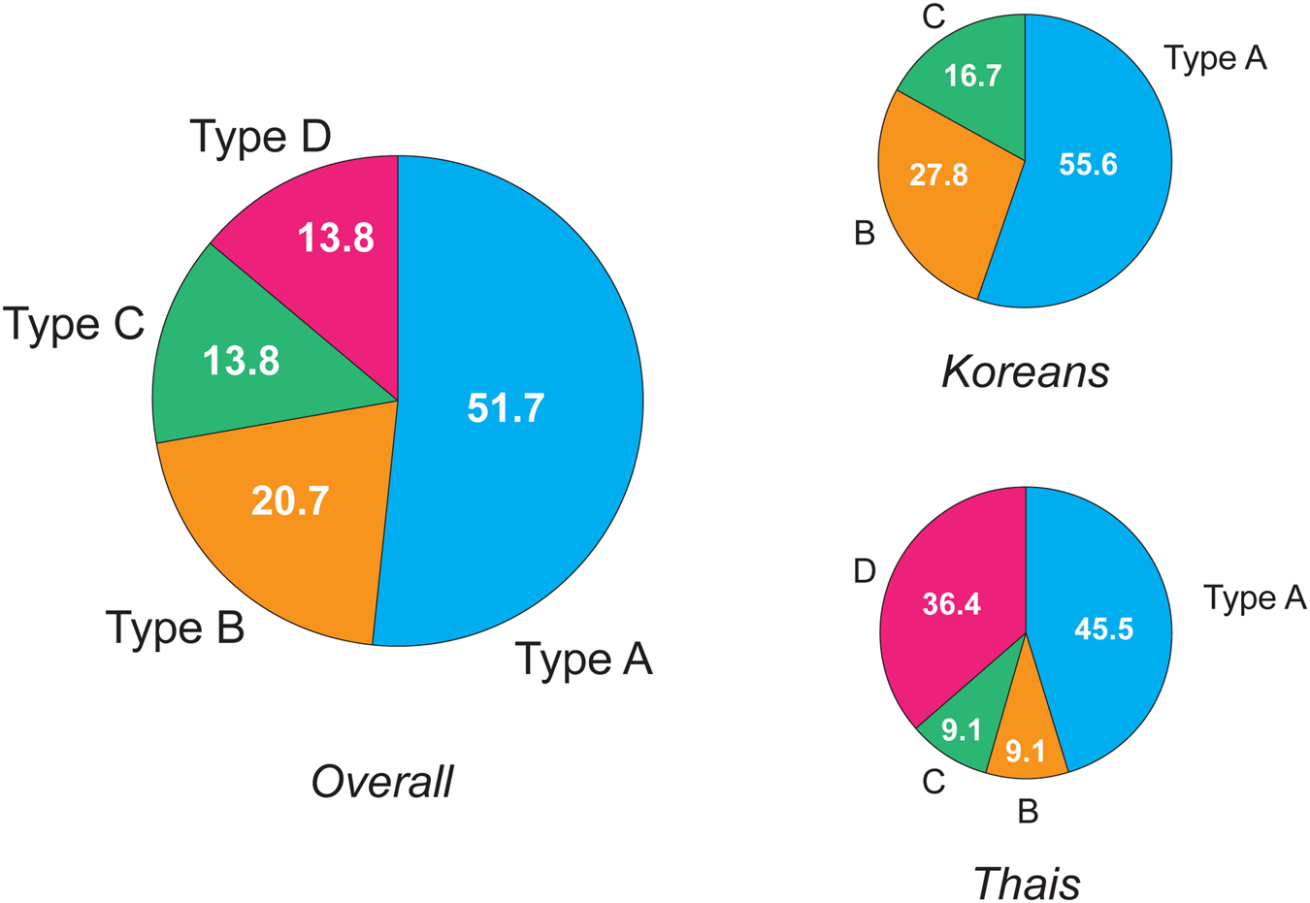
Proportion of the four patterns of innervation for the external branch of the superior laryngeal nerve (eSLN).

The piercing pattern of the eSLN to the CT or IPC was categorized into four types (Figs 5 and 6). The eSLN pierced the IPC only in type A specimens (15 cases, 51.7%). Independent ramifications of the eSLN pierced the IPC and CT in type B specimens (six cases, 20.7%). In type C specimens (four cases, 13.8%), the eSLN pierced the CT only. In type D specimens (four cases, 13.8%), the eSLN entered the CT after piercing the IPC, and this pattern was only observed in Thai specimens. Independent twigs entering the CT (types B and C) were observed superficially in about one-third of all cases.

**Figure 7:**
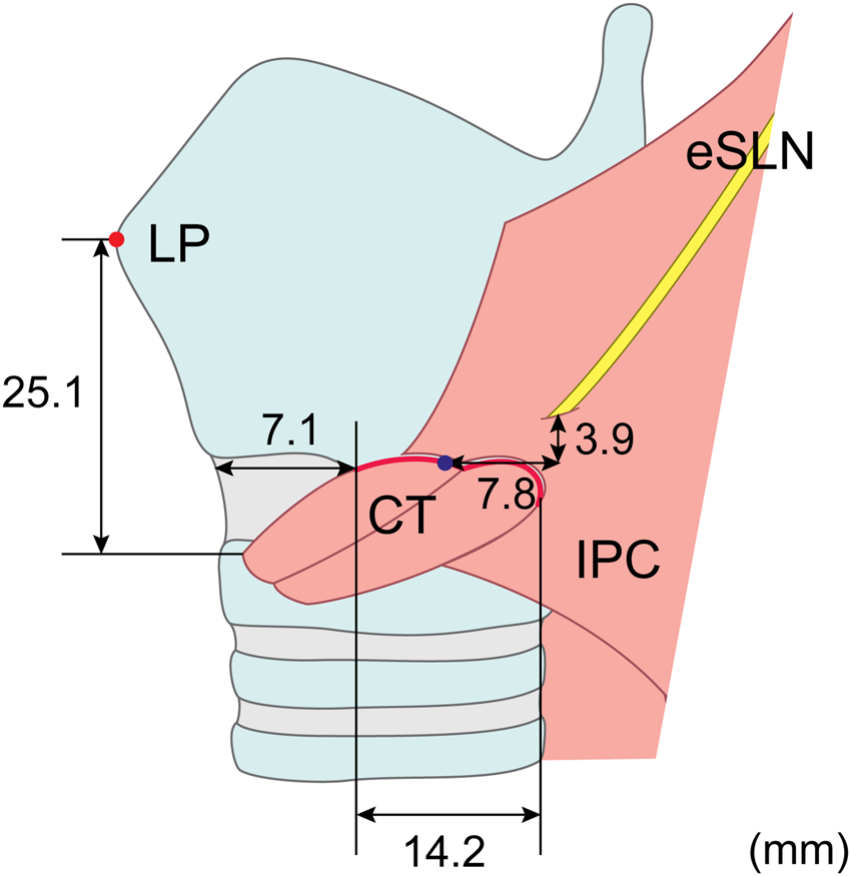
Location of the piercing point of the external branch of the superior laryngeal nerve (eSLN). LP, laryngeal prominence, IPC, inferior pharyngeal constrictor; CT, cricothyroid. IPC-CT joint was highlighted as the red line.

The horizontal length of the ICJ was 14.2 ± 3.6 mm (Fig. 7), with no statistically significant differences between Korean and Thai specimens (*p* > 0.05). The horizontal distance between the ICJ and the midsagittal line was 7.1 ± 3.6 mm, and this distance was greater in Thai specimens (8.4 mm) than in Korean specimens (5.9 mm) (*p* < 0.05). The distance from the PP to the midpoint of the ICJ was 8.7 ± 5.1 mm (7.8 mm posterior and 3.9 cm superior to the midpoint), and it was within 1 cm in 17 cases (57%).

## Discussion

The branches of the vagus nerve that innervate the muscles of the larynx are responsible for the meticulous control of muscular movements involved in respiration, deglutition, and phonation. Most of these muscles are innervated by the RLN, with the exception of the CT. Because RLN injury frequently results in dyskinesia of vocal ligaments and detrimental voice changes, many previous studies have investigated RLN anatomy to ensure proper identification and preservation of the structure during head and neck surgery^5–8^. In contrast, few studies have focused on the anatomy of the eSLN, although damage to this structure may also produce clinical symptoms, such as hoarse or breathy voice and a diminished range of vocal frequency. Considering its intimate topographic relationship to the STA and high injury rate (up to 58%), routine identification of the eSLN prior to surgical manipulation is necessary. However, a consistent, easily identifiable landmark for discerning eSLN anatomy remains to be identified. Therefore, in the present study, we examined the utility of three such landmarks—the STA, AP, and ICJ—for identification of the eSLN during a conventional surgical approach.

### Topographic relationship between the eSLN and STA

The branch of the STA that descends from the external carotid artery toward the upper thyroid complicates surgical approach to the carotid triangle, as damage to the STA frequently results in injury to the eSLN due to its topographic proximity to the STA. While the eSLN was a distinct nerve with the diameter of about 0.8 mm, rates of eSLN identification have varied greatly across previous studies ranging from 33% to 93%^6–8^. In the present study, we observed that the eSLN was medially juxtaposed to the STA in most cases (96.7%). However, in several cases, we observed that the eSLN pierced the posterior part of the IPC, making it difficult to locate twig-like branches in the vicinity of the CT. These IPC-covered portions of the eSLN may correspond to unidentifiable nerves described in previous studies. Friedman *et al*. also identified an anatomic variant in which the eSLN deeply innervated the IPC in 20% of cases, reporting that localization of the nerve was exceedingly difficult in this type^8^. Moreover, Cernea *et al*. reported that the level of surgical experience may be an important factor in ensuring preservation of the eSLN^3^. In their study, the injury rate was 28% when procedures were performed by residents, though only 12% when performed by the senior author (Claudio R. Cernea, MD).

We also observed that a tiny branch of the STA transversed the eSLN in 17% of cases in the present study. As this branch may be easily overlooked in comparison to the main trunk of the STA, there is a high possibility of vascular damage to the branch during identification of the eSLN.

Kierner *et al*. reported that the point of eSLN separation from the vagal nerve was located 2.9–5.6 cm from the carotid bifurcation^9^. However, detailed metric information regarding the topographic relationship between the eSLN and STA could not be obtained in the present study, since accidental displacement of these neuromuscular structures frequently occurs during dissection. If the eSLN were medially juxtaposed to the STA in the most cases, the PP may be more useful and consistent for eSLN identification. In the present study, the PP was located 1–2 cm anterior to the aECA. The point of STA ramification was situated slightly anterior to the aECA, and the horizontal distance from this point to the eSLN was approximately 1.5 cm in the anterior direction.

### Topographic relationship between the eSLN and the AP of the thyroid gland

The AP has been described as an important landmark for the identification of the eSLN. Previous studies have reported that the distance between the AP and eSLN is >1 cm in most Caucasians (42–62%)^3,9^. However, this pattern was observed in only one-quarter of Asian specimens in the present study, whereas the eSLN was situated within 1 cm of the AP in both Korean and Thai specimens. In addition, Ozlugedik S *et al*. reported the eSLN crossed the STA with 1 cm <distance from the AP in 60%^4^.

Cernea *et al*. reported that, among patients undergoing high-risk thyroidectomy, the eSLN crossed the vessels below the AP in 20% of cases, similar to the value of 21% observed in the present study^3^. Kirener *et al*. reported this pattern in 14% of cases^9^. In their study, cases in which the eSLN was located immediately above the AP were classified into another type (14%). This discrepancy may be explained by differences in the method of classification, as some cases of the latter type may be categorized into the high-risk group described by Cernea *et al*.

In the present study, the vertical distance between the AP and LP was approximately 3.5 cm, though a rather large standard deviation was observed (0.8 cm). Given that the dimension of the thyroid gland was not permanent, such a large range calls into questions whether the AP is an inappropriate landmark for identification of the eSLN. Interestingly, our statistical analysis (Pearson correlation analysis) revealed no statistically significant relationship between the vertical location of the AP and its distance to the eSLN. The vertical distance between the AP and the eSLN was approximately 0.8 cm, and this metric was highly concentrated around 1 cm, indicating that the AP may be a reasonable landmark for eSLN identification in specimens without abnormal dimensional changes to the thyroid gland (e.g., goiter). However, it should also be noted that, in some pathological conditions, superior displacement of the AP would decrease the distance between the AP and eSLN.

We also observed that the AP was innervated by tiny, twig-like branches of the eSLN in more than half of specimens in the present study. The eSLN is comprised of both efferent fibers that innervate the CT and afferent fibers that mediate sensation from the cricothyroid joint and membrane^9–14^. Since the physiological significance of the eSLN fibers to the thyroid gland was unknown, we were unable to determine whether this distribution of the eSLN is related to function of the thyroid gland, or whether injury to these twig-like branches leads to specific complications. Further studies are required to elucidate the clinical importance of these eSLN twigs to the AP.

### The ICJ as a landmark for eSLN identification

Although the eSLN provides only efferent fibers to the CT, its point of entry was not located at the CT in over half of cases in the present study. Lennquist *et al*. also reported that the eSLN pierced and remained covered by the uppermost fibers of the inferior constrictor in 20% of cases^15^.

The location of the PP seemed to be more consistent than the proceeding topography, indicating that the PP may be useful for eSLN identification during anterior surgical approaches, following reflection of the strap muscles. Similarly, the ICJ may also represent an adequate landmark for eSLN identification. Exposure of the ICJ can easily be achieved by a lateral reflection, with a 5 mm transection of the superomedial border of the sternothyroid^8^. In the present study, the horizontal distance of the ICJ from the midline was 8.4 mm in Korean and 5.9 mm in Thai specimens. Given that the ICJ length was approximately 1.5 cm in both races, the area 2 to 2.5 cm from the midline allows for surgical approach of the ICJ. Since the distance from the PP to the midpoint of the ICJ was approximately 1 cm, the PP can be easily identified when the posterior point of the ICJ is exposed.

A more complex pattern of eSLN topography was observed in Thai specimens: Type D, in which the eSLN entered the CT after piercing the IPC, was observed only in Thai specimens. With the exception of the horizontal distance of the ICJ to the midline, the locations of the ICJ and PP were not significantly different between Korean and Thai samples. However, a significant difference in the relative position of the PP to the ICJ was observed between Asian specimens of the present study and Caucasian specimens in previous studies. In a 20-year study involving 1,057 nerves of 884 patients, Freidman *et al*. reported that the eSLN could identified at the ICJ in 85.1% (n = 900) of patients^8^. In contrast, we observed that the PP was located posterior to the ICJ in more than half of cases in the present study.

Cernea *et al*. described a high-risk type, in which a portion of the eSLN covered by the IPC was located more than 10 cm anterior to the point of CT entry^3^. In contrast, the furthermost piercing point was located 2.5 cm from the midpoint of the ICJ in the present study. Cernea *et al*. also described a large positional deviation of the PP at the IPC, whereas the PP was located within 1 cm of this point in 56.7% of cases in the present study^3^. These findings indicate that the IPC-covered portion of the eSLN was more easily identifiable in Asian specimens of the present study than in Caucasian specimens of previous studies. Given that the PP was situated a short distance from the ICJ in our study, the ICJ may represent a consistent, reliable landmark for eSLN identification in Asian patients. However, it is important to note that identification of the PP near the ICJ cannot guarantee absolute preservation of the eSLN, as multiple twigs of the eSLN were encountered in the present study.

### The LP as a landmark for eSLN identification

The LP is the most remarkable structure of the laryngeal cartilages. Since the LP is superficially tangible, the position of the eSLN relative to the LP may aid in estimating the position of the PP prior to or during surgery. While the PP was located more anteriorly in Thai specimens than in Korean specimens, the distance of the PP from the aECA was greater in Korean (17.9 mm) than in Thai (13.6 mm) specimens. This paradoxical topography may have been associated with differences in the anteroposterior length of the neck.

The LP is generally located more anteriorly in adult men than in women, as this region becomes more prominent in men during puberty (Adam’s apple). Accordingly, the horizontal distance from the LP and PP was greater in men (27.5 mm) than in women (22.5 mm) of the present study. As this difference between the sexes is usually consistent, the LP may represent a valid landmark for eSLN identification, in which the PP in women is located 0.5 cm anterior to that in men.

### Neuroanatomical consideration of eSLN distribution

Previous neuroanatomical studies have revealed that the eSLN exclusively provides motor fibers to the CT, while other laryngeal muscles are controlled by the RLN. However, some studies have suggested that branches of the SLN participate in control of other laryngeal muscles, such as the thyroarytenoid and posterior cricoaryrenoid muscles (PCA)^11–14^. Hydman *et al*. reported that such branches of the SLN are associated with reinnervation and functional recovery of the PCA following injury to RLN axons^10^. In addition, their research provided electrophysiological evidence of alternative SLN innervation to the PCA in Sprague-Dawley rats: Retrograde tracing experiments revealed that a caudal group of PCA motor neurons projected to the RLN, while a rostral group projected to the SLN. Alternative pathways of SLN innervation may facilitate dual reinnervation to the laryngeal muscle following axonal injury of the RLN. In contrast, electromyographic experiments by Masuoka *et al*. indicated that the RLN stimulated the CT in approximately 40% of cases^16^. Anastomosis between the RLN and SLN branches, such as Galen’s anastomosis, may result in more complex patterns of vagal innervation in the larynx.

Folk *et al*. reported that isolated eSLN stimulation following RLN transection provided a glottic closing force with approximately 89% RLN control in a porcine model^5^. Although supplementary innervation of other laryngeal muscles by the eSLN may be involved in producing this glottic closing force, we cannot exclude the possibility that proprioceptive monitoring via the eSLN is involved in producing this protective response. The finding that the eSLN may possess both motor and sensory fibers is clinically relevant, as sensory fibers of the eSLN may play a role mediating vocal function^17–23^.

In the present study, the eSLN pierced only the CT, without twig-like branches to the IPC, in approximately 14% of cases (type C). In other types, eSLN twigs pierced only the IPC (type A) or both muscles. It is possible that, after piercing the IPC, the fibers may not have innervated the IPC in type A and C specimens of the present study. However, the pattern of innervation observed in type B specimens (20.7%, two independent twig-like branches entering the CT and IPC) suggests supplementary innervation of the IPC by the eSLN. Accordingly, anastomosis between the eSLN and RLN was also reported by Wu *et al*. and Sanders *et al*., who suggested that the eSLN may innervate laryngeal muscles other than the CT^24,25^.

Although additional neurophysiologic or histological studies are required to elucidate the relationship between eSLN distribution and motor function of the larynx, the anatomical importance of the PP and its involvement in laryngeal function suggest that the PP of the eSLN should be routinely identified in the vicinity of the ICJ during surgical procedures.

## Methods

All cadavers utilized in the present study were legally donated to Yonsei University College of Medicine in South Korea and Chulalongkorn University College of Medicine in Thailand. The present study was conducted in accordance with the Declaration of Helsinki. Thirty hemifaces were harvested from 16 embalmed cadavers (10 Korean and six Thai individuals; 12 men and four women; mean age: 80.5 years). The neck region was carefully dissected to reveal the topography of the eSLN, STA, AP, and laryngeal prominence (LP). During dissection, the samples were fixed using pins to avoid accidental displacement, and the topographic relationship between the eSLN and STA was observed.

We examined the following:

A. Location of the AP with reference to the LP.
B. Pattern of eSLN course to the AP and the vertical distance between them.
C. Location of the piercing point (PP) of the eSLN to the CT or IPC, with reference to the anterior border of the external carotid artery at the level of submandibular gland (aECA).
D. Location of the PP with reference to the LP.
E. Piercing pattern of the eSLN according to the muscles pierced (CT or IPC).
F. Horizontal length of the inferior constrictor – cricothyroid muscle joint (ICJ) and its horizontal distance from the midsagittal line.
G. Location of the PP with reference to the midpoint of the ICJ.

Distances observed in the present study were measured using Absolute Digimatic Digital calipers (range: 0–150 mm; catalogue no. 500-196-20; Mitutoyo, Kanagawa, Japan). Data were analyzed using PASW Statistics ver.18.0 (SPSS, Chicago, IL, USA) (${\rm{\alpha }}$ = 0.05). Differences in AP and eSLN location between sex and race groups were compared using *t*-tests. Pearson correlation coefficients were used to analyze differences in the vertical distance between the AP and LP, and between the AP and eSLN. The datasets generated during and/or analysed during the current study are available from the corresponding author on reasonable request.

### Data available statement

The datasets generated during and/or analysed during the current study are available from the corresponding author on reasonable request.

### Ethical approval

The methods were carried out in accordance with the 1964 Helsinki declaration and the cadavers were legally donated for the research by the Yonsei Universtiy College of Medicine and Chulalongkorn University College of Medicine.
